# Testing a new active learning approach to advance motor learning knowledge and self-efficacy in physical therapy undergraduate education

**DOI:** 10.1186/s12909-021-02486-1

**Published:** 2021-01-19

**Authors:** Daniela V. Vaz, Erica M. R. Ferreira, Giulia B. Palma, Osnat Atun-Einy, Michal Kafri, Fabiane R. Ferreira

**Affiliations:** 1 Department of Physical Therapy, Universidade Federal de Minas Gerais, Av. Pres. Antônio Carlos, 6627 - Pampulha, Belo Horizonte, MG 31270-901 Brazil; 2 Department of Physical Therapy, School of Physical Education, Physical Therapy and Occupational Therapy, Universidade Federal de Minas Gerais, Belo Horizonte, Brazil; 3 Department of Physical Therapy, Faculty of Social Welfare and Health Sciences, University of Haifa, Haifa, Israel

**Keywords:** Motor learning, Active learning, Education, Physical therapy

## Abstract

**Background:**

Motor learning (ML) science is foundational for physical therapy. However, multiple sources of evidence have indicated a science-practice gap. Clinicians report low self-efficacy with ML concepts and indicate that the lack of access to systematic training is a barrier for practical implementation. The general goal of this preliminary study was to describe the effects of a new educational intervention on physical therapy student’s ML self-efficacy and knowledge.

**Methods:**

Self-efficacy was assessed with the Physical Therapists’ Perceptions of Motor Learning questionnaire. Data was acquired from third-semester students before their participation in the ML educational intervention. Reference self-efficacy data was also acquired from physical therapy professionals and first and last-semester students. The educational intervention for third-semester students was designed around an established framework to apply ML principles to rehabilitation. A direct experience, the “Learning by Doing” approach, in which students had to choose a motor skill to acquire over 10 weeks, provided the opportunity to apply ML theory to practice in a personally meaningful way. After the intervention self-efficacy was re-tested. ML knowledge was tested with an objective final exam. Content analysis of coursework material was used to determine how students comprehended ML theory and related it to their practical experience. The Kruskal-Wallis and Mann-Whitney U tests were used to compare self-efficacy scores between the four groups. Changes in self-efficacy after the educational intervention were analyzed with the Wilcoxon test. Spearman rank correlation analysis was used to test the association between self-efficacy and final exam grades.

**Results:**

By the end of the intervention, students’ self-efficacy had significantly increased (*p* < 0.03), was higher than that of senior students (*p* < 0.00) and experienced professionals (*p* < 0.00) and correlated with performance on an objective knowledge test (*p* < 0.03). Content analysis revealed that students learned to apply the elements of ML-based interventions present in the scientific literature to a real-life, structured ML program tailored to personal objectives.

**Conclusions:**

Positive improvements were observed after the intervention. These results need confirmation with a controlled study. Because self-efficacy mediates the clinical application of knowledge and skills, systematic, active training in ML may help reduce the science-practice gap.

**Supplementary Information:**

The online version contains supplementary material available at 10.1186/s12909-021-02486-1.

## Background

The centrality of movement science, including motor learning (ML), in physical therapy is widely accepted [1, 2] yet translating scientific theory to practice is limited. A survey of 289 physical therapists (PTs) found recognition of the importance of ML [3]. PTs may implicitly apply several ML strategies in practice but may have limited ability to incorporate ML knowledge coherently in their clinical reasoning. Think-aloud interviews about videotaped neurologic rehabilitation sessions have shown that PTs do implement various ML components [4]. However, another study revealed that PTs had only a partial and incoherent understanding of ML’s theoretical concepts and ML-based interventions [5]. The PTs failed to link their understanding to specific, practical actions with patients, and had difficulty articulating and distinguishing knowledge of ML from other constructs. Additionally, they lacked confidence in their own ML knowledge, suggesting that their treatment choices were “guided by intuition” [5]. ML is often used as a catch-all term, which adds to confusion about the theory and its application [6]. These findings agreed with other studies [7, 8] showing inconsistent use of terminology and a need to enhance clinicians’ self-confidence. The lack of systematic and accessible ML training appears to be the main barrier to ML-based clinical practice [3, 5, 9].

PT professional curriculum guidelines recommend including ML learning experiences [2]. Exposure to applied knowledge during the early stages of training increases the likelihood of that knowledge being used later in clinical practice [10, 11]. Therefore, early, active learning strategies should be introduced so that students can directly experience ML situations, apply theory-based reasoning to solve practical problems, and consolidate their skills for clinical practice.

ML science has not, however, been consistently incorporated in PT education. ML knowledge is complex and multidisciplinary, with a wide scope of elements not uniformly defined or distinguished from one another [4, 6, 8]. Therefore, concrete recommendations for ML application in clinical practice (i.e., ML frameworks) are rare. Fortunately, two recently published tools can be useful for structuring ML education for PTs.

The Physical Therapists’ Perceptions of Motor Learning (PTP-ML) allows the education program to be tailored to practitioners’ ML perspectives. The PTP-ML is a standardized questionnaire with adequate construct validity, internal consistency, and test-retest reliability [3] that assesses PTs’ self-efficacy, self-reported ML implementation, and environmental workplace factors affecting implementation with three separate scales [3]. Self-efficacy refers to one’s beliefs about his/her capabilities to successfully perform a particular behavior or task [3]; it mediates the implementation of knowledge and skills in clinical practice [12, 13] because competent performance requires not only knowledge and skills but also belief in one’s personal ability to use both effectively. With the self-efficacy scale of PTP-ML, educators can map gaps in clinicians’ and students’ ML self-efficacy before and after educational interventions.

**Fig. 1:**
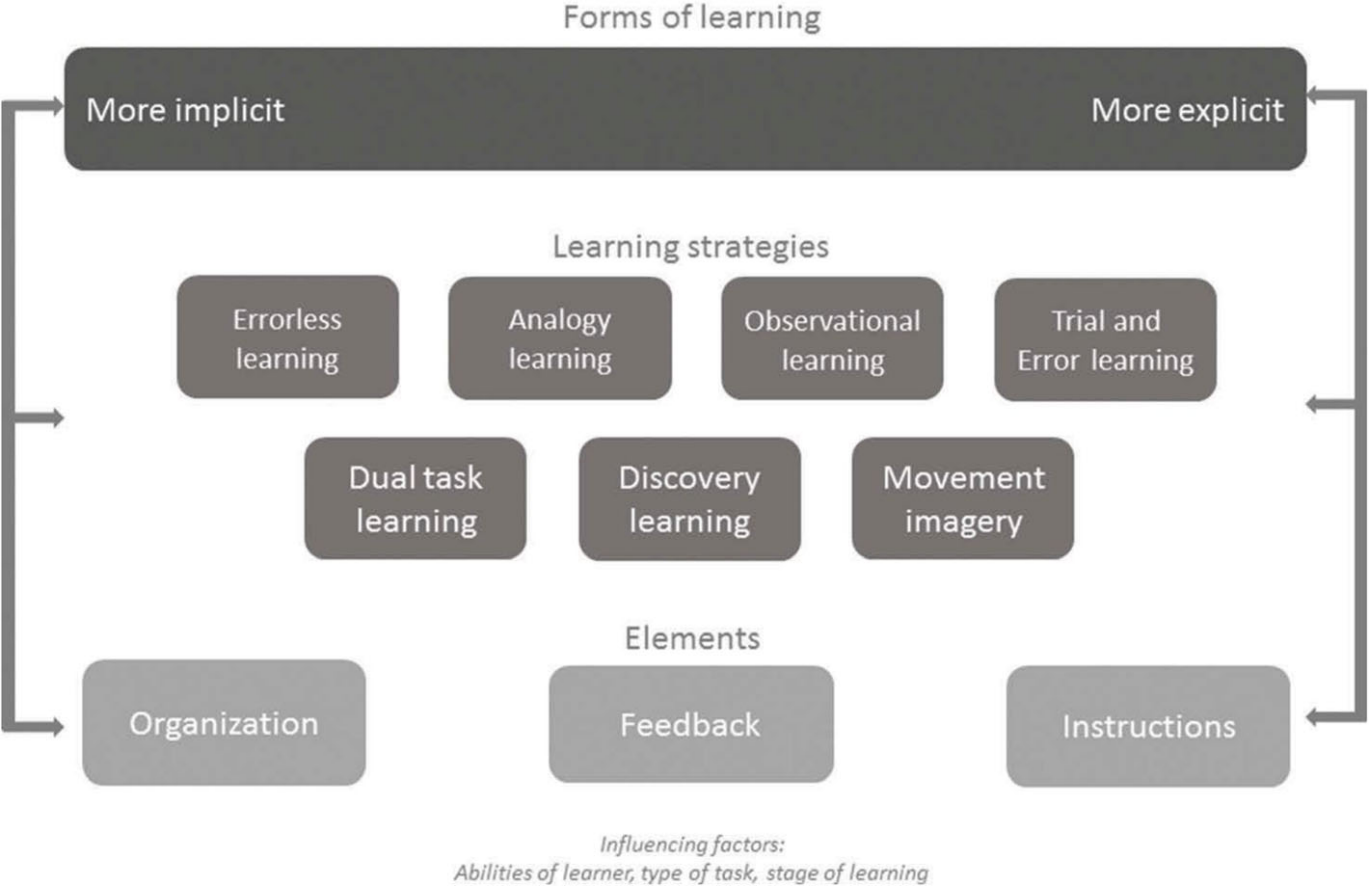
A framework for the application of motor learning by health-care professionals, Kleynen et al. (2018). Reprinted with permission of Taylor & Francis in line with Scientific, Technical, Medical (STM) publishers’ agreement

The second tool is a framework by Kleynen et al. [14] that integrates scientific research and expert knowledge to support ML application in clinical practice. It is organized into three “layers” of decisions to be made for individual patients (Fig. 1). The most general layer provides an overarching classification of forms of learning—implicit or explicit. The next layer presents several learning strategies to choose from, including Analogy, Errorless, Trial and Error, Imagery, Discovery, Dual-task, and Observational learning. The final layer pertains to decisions about fundamental ML elements: practice organization, instructions, and feedback. Therapists consider patients’ abilities, type of motor task, and learning stage when making choices within the framework. Case examples and extensive supporting literature accompany the framework. It provides a taxonomy and overview to assist well-informed decisions concerning ML [14].

Given the centrality of ML for PT practice, the gaps in ML education among PTs, and the benefits of early training, this paper reports a preliminary investigation of an early, active ML educational intervention planned to provide students with structured knowledge and tools to support well-reasoned, tailored use of ML principles in daily contexts. The educational intervention is innovative because in most PT programs ML contents are not taught in a structured and dedicated course but are instead distributed unsystematically over several different courses [15, 16]. The general goal of this study was to investigate the results of the educational intervention on student’s ML self-efficacy and knowledge. Specific aims were to (1) describe and compare self-efficacy of students at the beginning and at the end of the PT program and experienced PT professionals, (2) compare self-efficacy before and after the educational intervention, (3) compare self-efficacy after the educational intervention to that of reference groups who have only received conventional ML education (4) test the association between self-efficacy after the educational intervention and an objective knowledge measure, and (5) explore how students applied theoretical ML concepts to their experience of active learning.

## Methods

### Study design

This was a quasi-experimental study with one intervention group and three reference groups. ML self-efficacy was investigated for all four groups and results were used to plan an ML educational intervention. The intervention group participated in the ML course and took a second ML self-efficacy assessment and an objective ML knowledge test after the course. The student’s coursework was described with content analysis. Procedures were approved by the local Ethics Committee. All participants provided written informed consent to the use of their data for research purposes.

### Participants

PTs with ≥5 years’ experience in various fields (sampled by convenience) and students (purposive samples) at a leading PT education institution in Brazil (which offers PT programs at the baccalaureate level over 10 semesters) were invited to participate in the study. They comprised an intervention group of 28 third-semester students (age 21.6 ± 5.67, 7 males) and three reference groups: (1) 40 first-semester students (age 20.8 ± 3.03, 7 males), (2) 31 last-semester students (age 24.7 ± 2.3, 7 males), and (3) 29 PTs (age 36.8 ± 6.97, 8 males). All third-semester students enrolled in the 60-h ML course (i.e. intervention). Last-semester students’ and professionals’ levels of self-efficacy were expected to reflect the effects of the conventional practice of teaching ML unsystematically, with contents distributed over several courses. A lecturer (DVV) invited the participants to answer the PTP-ML. Their data was used to prepare the course.

### Instruments and procedures

#### Self-efficacy assessment

To assess students’ and clinicians’ ML-related self-efficacy, the original self-efficacy scale of the PTP-ML was translated by two independent PT professors to Portuguese and then back-translated to English by two independent native English speakers; a committee comprised by two PT professors and a clinician examined the original, the Brazilian and the back-translated versions to ensure they had semantic and conceptual correspondence. The final version was approved by the original authors. An adapted version of the questionnaire suitable for assessing and facilitating ML education in undergraduate PT programs was designed that consisted of parts A and B (available in Fig. 4). Part A contains the 12 self-efficacy statements from the original PTP-ML and covers self-assessed knowledge and the ability to explain ML principles or terms [3]. Part B was created for this study and contains eight self-efficacy statements pertaining to the content of the framework by Kleynen et al. [14]. The five response options (Likert scale) vary from strong disagreement [1] to strong agreement [5] with self-efficacy statements. The final score of each scale is the average of all item responses within that scale so that overall scale scores below 3 (the neutral point) indicate low self-efficacy, and above 3 indicate high self-efficacy. A sample of 45 students and 19 professionals (who did not take part in the present study) took the adapted version twice (7 days apart) for reliability analysis. The Intraclass Correlation Coefficients (two-way mixed, single measures, for absolute agreement) were 0.92 and 0.88 and Cronbach’s Alphas were 0.93 and 0.92 for parts A and B, respectively. The smallest detectable change (SDC) values (the square root of the mean square error term from the ICC ANOVA [17]) were 1.38 and 1.57 and the limits of agreement (LOA) [17] were 0.78 and 0.99 for Parts A and B, respectively. For part A the authors of the original instrument (O. A. & M. K.) consider that the minimal important change [18] is 2.1, and for part B we consider the same value, because improvements that exceed this value are sufficient to change the lowest possible score indicating no ML self-efficacy (score of 1) to a score indicating some ML self-efficacy (3.1).

#### ML course

The 60-h ML course (see course description in the supplementary material) included lectures based on the framework by Kleynen et al. [14] with special emphasis given to the themes showing lower self-efficacy on the PTP-ML among the 128 participants of this study. The framework was presented to the third-semester students on the first day of class as their learning goal. They were informed that all lectures and readings (i.e., textbook chapters and additional scientific papers) would refer to the framework layers and components; after the course, they would be knowledgeable about its contents.

An active learning methodology was also employed. Since the third-semester students were not in any clinical placements, a direct experience approach to teach ML principles was implemented in the “Learning by Doing” [19] project. This approach has been successfully implemented in graduate-level skill acquisition courses [19, 20]. Each student chose a motor skill of their preference (e.g. dance styles, musical instruments, sports, circus skills, etc.) to acquire and practice for 10 weeks so that students experimented with ML strategies within their own experience. The students had to define a performance goal for 10 weeks, choose performance measures to track progress during that period, practice 3–8 h per week, maintain a log, participate in biweekly discussions about their ML efforts, write a paper about their chosen skill with recommendations for learning it, and demonstrate their level of skill on the last day of class. The students used content from Kleynen et al.’s framework [14] to plan and analyze their motor skill acquisition.

The students recorded their experience in five online, biweekly forms with open questions. The questions pertained to training volume and structure, performance goals, performance measurements and results, factors interfering with training, plans and goals for the next 15 days, and connections made between theoretical concepts and practical experience. Student answers were automatically saved in a spreadsheet and submitted to content analysis.

At the end of the course, a final exam with 42 multiple-choice questions including a clinical case (see case in supplementary material) designed to test content related to the PTP-ML parts A and B (ML framework [14]) was given. All but two students retook the PTP-ML before taking the final exam. After the course ended, the students were informed about the study and invited to provide their written coursework (exams and online forms) for analysis. Those who agreed (all 28 students) provided written informed consent. Data collection took place during the first semester of 2019.

### Data analysis

PTP-ML baseline scores were compared between the four groups (aims 1 and 3) using the Kruskal-Wallis test, as the Shapiro-Wilk test indicated that some scores were not normally distributed. The Mann-Whitney U test with Bonferroni correction was used to find pairwise differences (ɑ = 0.05 was divided by six possible comparisons leading to a 0.008 corrected level of significance). The Wilcoxon signed-rank test was used to examine differences in PTP-ML scores before and after the course (aim 2). Spearman correlation analysis was used to test the association between PTP-ML scores and ML knowledge (final exam grades) (aim 4). Significance levels were set at ɑ = 0.05.

Content analysis [21] was used to describe the content of biweekly logs in terms of well-defined codes in a systematic and replicable manner (aim 5). In content analysis codes may be predetermined from the literature or emergent from the data; a combination of both was used in this study [21]. Predetermined ML codes were based on a literature review conducted by two of the authors [15]. This review identified conceptual frameworks for applying ML knowledge to physical therapy and rehabilitation practice and established 25 ML elements from those frameworks. The elements included those governing the learning process and informing clinical practice (e.g., stages of skill acquisition, implicit or explicit learning mechanisms, meaningful goal setting, active involvement, etc.), elements operationalizing practice (e.g., type of feedback and order of practice), and elements referring to intervention strategies or specific methods (e.g., task-specific or observational learning). Emergent codes were based on the researchers’ experience as the teacher (DVV), teaching assistant (EM), or former student (GP) of the ML course. These codes were added using an iterative process and were documented in an analytical log (see the code definitions in the supplementary material).

To assess the reliability of the coding process, a sample of the data (10% of the answers to the biweekly forms’ questions) was coded independently by an experienced physiotherapist working as a teaching assistant in the ML course (EM) and by a research assistant (GP) who had taken the course. Excellent agreement was found between coders (kappa averaged for all codes = 0.928, 95% confidence interval 0.88–0.98).

## Results

### Between-groups comparison of self-efficacy scores before the educational intervention (aim 1)

**Fig. 2:**
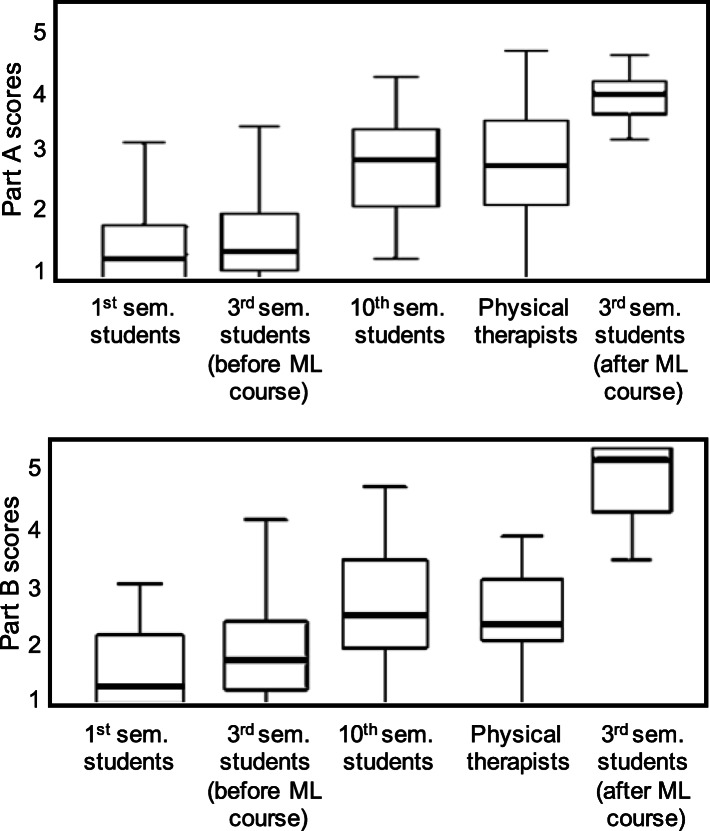
PTP-ML scores for all respondents and third-semester students after the ML course

Boxplots of PTP-ML scores for all groups are shown in Fig. 2. Before the ML course, self-efficacy scores from part A and the results from part B were significantly different between groups (*H* [3] = 50.56, *p* < 0.05 and *H* [3] = 27.42, *p* < 0.05, respectively).

The part A scores of professionals (*Mdn* = 2.83 [1.00, 4.67]) and last-semester students (*Mdn* = 2.92 [1.33, 4.25]) did not differ (*U* = − 0.93, *z* = − 0.10, *ns*). Scores of first-semester (*Mdn* = 1.33 [1.00, 3.42]) and third-semester students (*Mdn* = 1.46 [1.00, 3.75]) also did not differ (*U* = − 8.84, *z* = − 0.96, *ns,* respectively). First- and third-semester scores were lower than those of last-semester students and professionals (first compared to last: *U* = − 49.75, z = − 5.70, *p* = 0.00, *r* = − 0.68; third compared to last: *U* = 40.91, z = 4.22, *p* = 0.000, *r* = 0.56; first compared to professionals: *U* = 48.82, z = 5.49, *p* = 0.00, *r* = 0.66; third compared to professionals: *U* = 39.98, z = 4.06, *p* = 0.00, *r* = 0.55).

Regarding part B, professionals (*Mdn* = 2.25 [1.00, 3.63]) and last-semester students (*Mdn* = 2.38 [1.00, 4.38]) did not differ (*U* = − 4.35, *z* = − 0.46, *ns*). Last-semester students had higher scores compared to third-semester (*Mdn* = 1.69 [1.00, 4.63]; *U* = 25.83, *z* = 2.68, *p* = 0.007, *r* = 0.35) and first-semester students (*Mdn* = 1.00 [1.00, 2.88]; *U* = − 39.80, *z* = − 4.59, *p* = 0.00, *r* = − 0.55). The scores of first- and third-semester students did not differ (*U* = − 13.97, *z* = − 1.53, *ns*).

### Comparison of self-efficacy scores before and after the educational intervention (aim 2)

After the ML course, third-semester student scores significantly increased for Part A (*Mdn* = 1.46 [1.00, 3.75] to *Mdn* = 3.96 [3.25, 4.58]; *z* = − 2.81, *p* = 0.005, *r* = − 0.39) and part B (*Mdn* = 1.69 [1.00, 4.63] to *Mdn* = 4.81 [3.25, 5.00]; *z* = − 2.27, *p* = 0.02, *r* = − 0.31). The changes for Parts A (2.5) and B (3.12) were above the SDC, LOA and minimal important change values.

### Between-groups comparison of self-efficacy scores after the educational intervention (aim 3)

Significant group differences were also found for part A (*H* [3] = 75.13, *p* < 0.05) and part B scores (*H* [3] = 75.58, *p* < 0.05). Pairwise comparisons showed that students who took the ML course had significantly higher part A (*Mdn* = 3.96 [3.25, 4.58]) and part B scores (*Mdn* = 4.81 [3.25, 5.00]) than both last-semester students (*Mdn* = 2.92 [1.33, 4.25]; *U* = − 38.60, z = − 3.98, *p* = 0.00, *r* = − 0.53; *Mdn* = 2.38 [1.00, 4.38]; *U* = − 48.13, z = − 4.98, *p* = 0.00, *r* = − 0.65, respectively) and professionals (*Mdn* = 2.83 [1.00, 4.67]; *U* = − 38.25, z = − 3.88, *p* = 0.001, *r* = − 0.52; *Mdn* = 2.25 [1.00, 3.63]; *U* = − 52.06, z = − 5.30, *p* = 0.00, *r* = − 0.71, respectively).

### Correlations between self-efficacy scores and final exam performance (aim 4)

**Fig. 3:**
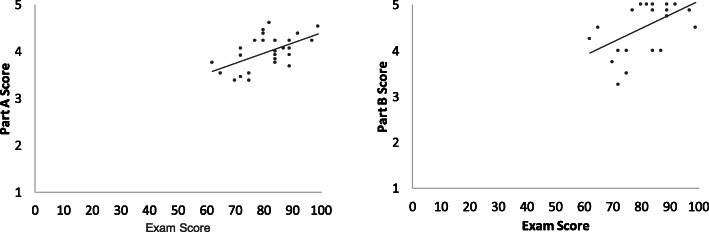
Scatter plots showing the correlation between self-efficacy scores and performance on the final exam

**Fig. 4:**
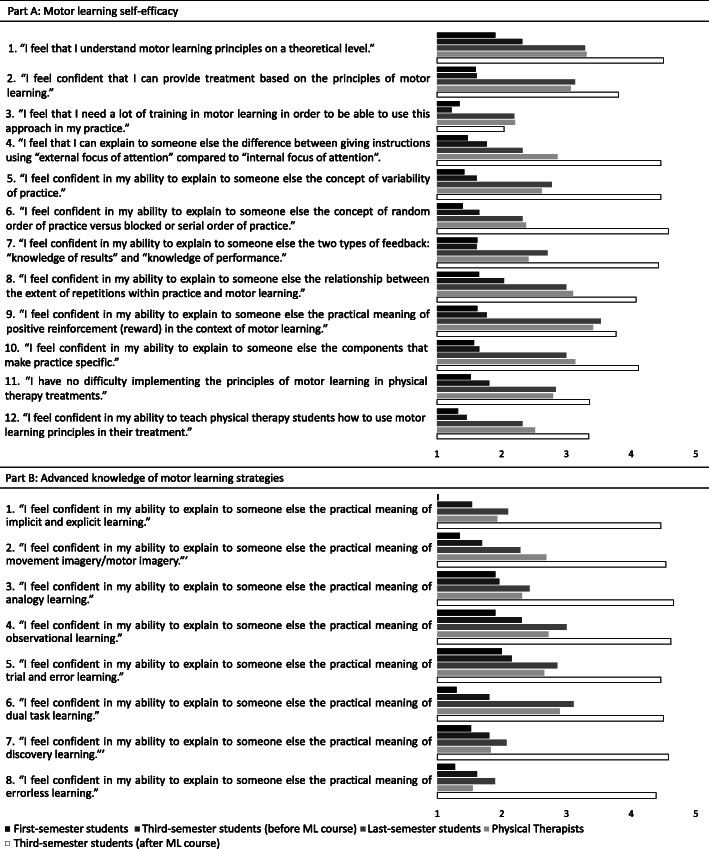
Scores of each PTP-ML item averaged over participants of each group

After the course, the final self-efficacy scores for parts A and B were significantly correlated with final exam grades (*rho* = 0.49, *p* = 0.01 and *rho* = 0.44, *p* = 0.02, respectively; Fig. 3). Figure 4 shows the average score for each PTP-ML item for the participants of each group.

### Qualitative data: content analysis of student’s reports of their experience of active learning (aim 5)

For the Learning by Doing project, the students chose skills related to dance (belly dance, samba), musical instruments (guitar, ukulele, piano, keyboard), sports (kung fu, soccer, skating, roller skating, slackline), exercise (gymnastics, Pilates, yoga, handstand), circus (juggling), art (macramé, calligraphy), and work (typing). The rate of completion for the five biweekly forms was high (100, 96, 93, 89, and 93% from the first to last, respectively); 132 forms were submitted. The average reported training volume was above the recommended 6 h per fortnight minimum (7:21, 6:39, 6:33, 6:19, first to fourth), except on the last form (5:10).

Content analysis of the forms showed how frequently each ML element was used by students during their motor skill acquisition (Table 1). All 25 predetermined ML codes were identified in the logs except the codes “task-oriented/task-specific training” and “specificity of practice.” The most frequently utilized ML elements were “stages of skill acquisition,” “focus attention,” “content and type of feedback,” and “task breakdown.” “Learning mechanism,” “order of practice,” and “observational learning” were also frequently used. Codes referring to “transfer of learning” and to mistakes in applying specific concepts were added to capture content not covered by the predefined codes, but the frequency of mistakes was generally low. On average, students mentioned 13 ± 7.3 codes in their forms (see Table 1 for exemplary quotes for each code).

**Table 1:** Frequency of motor learning codes in each biweekly log and exemplary quotes

Motor learning element	Biweekly log					Total	Exemplary quotes
	1	2	3	4	5		
1. Stages of skill acquisition	11	11	9	10	6	**47**	“The initial stage is indeed ‘very cognitive’ because it demanded a lot of my attention and memory, and my movement was very fragmented, with very unstable performance and several mistakes.” (G. M. Juggling, Form 1)”
2. Goal setting, meaningful goals	1	2	4	4	3	**14**	“By the end of two weeks, I want to be able to walk 4 m on the slackline (30-40 cm above the ground) in 90 s, with no falls and occasional external support” (I. S. Slackline, Form 3).
3. Active involvement/ problem solving	2	3	4	2	0	**11**	“I identified some problems caused by typing faster than necessary. Too much speed causes mistakes...I tried to find a speed that is more adequate to my current skill level” (A. F. Typing, Form 4)
4. Challenge /task difficulty	2	3	2	3	1	**11**	“It was very hard for me to accomplish my fortnight goal (because of the complexity of the musical piece itself). Therefore, I had to practice more than I expected” (A. M. Piano, Form 2)
5. Learning mechanism (implicit/explicit)	2	7	4	3	4	**20**	“I noticed that I am using a more explicit form of learning, because I am aware of what I need to do and I memorize facts and rules about the movement” (J. B. Guitar, Form 1)
6. Classification of motor skills/type of task	2	1	4	3	2	**12**	“...I classified my skill as gross, discrete, closed, simple and self-paced” (L. B. Belly dance, Form 3)
7. Content and type of feedback	2	6	7	8	6	**29**	“My instructor was using prescriptive feedback, like “you should have put your hand more to the right “(A. T. Gymnastic, Form 3)
8. Frequency and/or timing of feedback	1	3	1	3	0	**8**	“I was getting extrinsic feedback after I played the chords, it helped me to adjust my movements and improve performance.” (C. F. Keyboard, Form 4)
9. Focus attention (internal/external)	9	11	8	10	7	**45**	“This fortnight I used the concepts of internal and external focus a lot. The internal focus helped me perfect the movements that I was already comfortable with … The external focus was useful for transfer movements. Because I was afraid of performing them, I tried to focus more on the place where my body should fall, on the wall in front of me, and less on the movement itself” (A. T. Gymnastic, Form 3)
10. Task breakdown (whole/part)	7	7	4	5	4	**27**	“I have realized that I am using a progressive part method for practice, because as I learn parts of the skill I then add new parts, this helps my memory” (J. B. Guitar, Form 1)
11. Amount of practice	2	2	1	3	0	**8**	“I wrote the same sentence 9 times at each practice session” (G. C. Artistic calligraphy, Form 1)
12. Practice variability (constant/variable)	3	3	2	1	0	**9**	“Varied practice (practicing musical pieces other than my goal-song) has been helping me to refine my learning” (J. B. Guitar, Form 1)
13. Order of practice (random/serial/block)	3	5	6	4	2	**20**	“This week I used the blocked practice concept. I began making new bracelet model only after I had finished the previous one” (A. F. Macrame, Form 2)
13b. Order of practice conceptual mistakes	1	0	0	0	0	**1**	“I used random practice: I wrote the same letter four times, a different letter another four times and alternated them until I completed 16 repetitions for each letter” (G. C. Artistic calligraphy, Form 1)
14. Practice distribution (massive or distributed)	3	3	4	5	1	**16**	“I am using distributed practice, because it is best for retention” (A. P. Piano, Form 3)
15. Specificity of practice	0	0	0	0	0	**0**	
16. Positive reinforcement	0	1	2	2	0	**5**	“In addition to positive reinforcement, when I nail it, he hugs me and celebrates with me.” (N. C. Handstand, Form 4)
17. Task-oriented/task specific training	0	0	0	0	0	**0**	
18. Mental practice	4	2	2	4	4	**16**	“While I rested, I did some mental practice, imagining the positions and ways to improve them” (C. F. Keyboard, Form 3);
19. Manual guidance	0	0	1	0	0	**1**	“I asked my boyfriend to help me get into the correct position, because I couldn’t do it alone. I could complete the movement with his help” (N. C. Handstand, Form 3)
20. Observational learning/modeling	3	3	2	3	9	**20**	“I kept attempting to learn through observation video recordings of the movement” (C. S. Samba, Form 4)
21. Dual-task learning	0	2	3	0	0	**5**	“I tried to use dual task learning, in which I should execute the movement while talking to someone” (A. T. Gymnastic, Form 3)
22. Discovery learning	0	0	0	0	0	**0**	
23. Trial and error learning	1	3	1	2	3	**10**	“I first tried trial and error learning, so that I could better observe what mistakes I was making (there were many in this fortnight)” (A. M. Piano, Form 2)
24a. Errorless learning	0	0	1	1	0	**2**	“When certain hand or finger movements were very hard for me, I choose blocked practice to decrease the erros.” (A. P. Piano, Form 3)
24b. Errorless learning conceptual mistakes	1	1	2	1	0	**5**	“I tried to practice all the chords with making as few errors as possible, always correcting them to achieve a better result” (C. F. Keyboard, Form 3)
25. Analogy learning	0	0	0	0	1	**1**	“The analogy be able to dance the samba was to “draw a heart on the floor with the feet” (C. S. Samba, Form 4)
26. Transfer of learning	2	2	0	2	0	**6**	“I used the concept of transfer. I am walking on curbs, hoping to improve my balance for when I begin to practice on the slackline” (I. S. Slackline, Form 1)

## Discussion

This quasi-experimental study was the first to describe an early, active learning approach promoting ML knowledge and self-efficacy in undergraduate PT education. This unique educational intervention was built from systematic, clinically framed ML knowledge and a combination of theory and practice. The course was a first effort to help close the ML science-practice gap [3, 22–24]. After the course there was a significant and relevant increase in PT students’ ML self-efficacy that correlated with performance on a knowledge test. Content analysis showed that the learning project offered rich, varied opportunities to experiment with fundamental ML concepts described in the scientific literature.

### Self-efficacy before the educational intervention

Students’ and professionals’ self-efficacy mediates the implementation of knowledge and skills in clinical behavior [12, 13], and low self-efficacy may contribute to the ML knowledge-practice gap. Before the ML course, last-semester students’ self-efficacy was statistically higher than that of first- and third-semester students. However, last-semester students’ scores on parts A and B were below 3 (2.92 for part A and 2.38 for part B), indicating that they mostly did not agree with statements affirming ML self-confidence. These findings, consistent with reports on PT programs in other countries [15, 16], were probably related to the nature of the PT program, in which ML content was scattered over applied courses (especially pediatric and neurologic rehabilitation). This may explain the insufficient increase in self-efficacy between first and last-semester students (1.59 and 1.38 for parts A and B, below the minimal important change of 2.1). Additionally, the self-efficacy of professionals with ≥5 years of clinical experience (2.83 for part A and 2.25 for part B) did not differ from that of last-semester students; it was equivalently low (below 3, indicating low self-efficacy). This finding may be due to the lack of continuous education. Similar low self-efficacy (average 2.95 ± 0.7) was reported in a previous study of PTs practicing in Israel [3], where the PTP-ML questionnaire was developed. These findings confirmed that systematic educational activities requested by professionals are indeed needed [15].

### Early and active educational strategy

The ML course was offered early in the PT program to increase the likelihood of application in clinical practice [11]. It was based on active, experiential engagement with ML content; students spoke and wrote about the motor skills they were acquiring, related their motor learning process to past experiences, and applied their newly acquired knowledge to their daily lives [25]. This provided a foundation for professional development in which ML is integral.

The students practiced a variety of skills, and their experiences were similar to that reported by van der Kamp, Withagen, and Ort, ([20], p. 5) who also teach a perceptual-motor learning course with an experiential approach:

“Along the way, they actively explore the affordances of skill learning: they move, imitate, try, expose themselves to errors, repeat, feel, correct, take risks, get energized, quit, plan, reflect, vary practices, think, get bored, frustrated, are proud, notice and adapt; in short, they . . . become attentive to increasingly differentiated aspects of learning.”

Due to this rich experience, many students expressed gratitude to the course instructor (DVV) for the opportunity to “put themselves in the patient’s shoes” and develop their affective skills.

The teacher’s role was not transmitting knowledge but rather offering timely feedback and opportunities for students to create personal, new knowledge [20, 26]. This active approach to learning attempted to reach the upper level of Bloom’s taxonomy (Creating); students combined ML elements to form a functional whole, they reorganized the elements into a unique, personalized structure through *generating* learning goals, *planning* measurements and training programs, and *producing* their own skill acquisition experience [27].

Content analysis focused on identifying the fundamental elements of ML-based interventions in student reports. The elements included theoretical concepts, practice variables, and intervention strategies representative of several conceptual frameworks for applying ML knowledge to PT practice published from 2000 to 2017 [15]. The content of the biweekly forms indicated that all but two elements, “task-oriented/task-specific training” and “specificity of practice,” were used. As task-specific practice was built into the Learning by Doing a project, its mention was unnecessary. Overall, the content analysis suggested that the course provided plentiful opportunities for firsthand experience putting ML theory into context. Mistakes were very few, suggesting that the course structure and method were effective to promote solid ML knowledge.

### Changes in self-efficacy after the educational intervention

After the course, the students’ median self-efficacy scores rose to 3.96 (part A) and 4.81 (part B), indicating that they agreed or strongly agreed with statements affirming ML self-confidence. The observed increases (2.5 for Part A and 3.12 for part B) were above the SDC and LOA values, indicating true change beyond that of measurement error, and also above the minimal important difference, indicating relevant improvement. After the course, student’s self-efficacy was superior to that of last-year students and professionals who had been exposed only to the conventional practice of teaching ML contents spread unsystematically over several courses. We cannot, however, definitely attribute the observed improvements to the intervention, as there was no true control group with random allocation of participants. Future controlled studies are necessary to confirm the findings of this quasi-experimental study.

Self-efficacy scores after the course were moderately correlated to performance on the knowledge test. The observed correlation values are in very close agreement with the estimated overall correlation of 0.49 between self-efficacy measures and grade point average provided by a meta-analysis including data from 9598 students [28]. This is an important finding. First, the close agreement with previous evidence suggests good validity for both the self-efficacy measure and the knowledge test. Second, the finding that ML self-efficacy did not vary independently of an objective knowledge test after the ML course was a positive result because self-efficacy is a subjective judgment of capabilities. A lack of correlation would indicate a subjective appraisal of competence with no grounding on actual knowledge and skills [29], which could hinder the efficacy of health professionals.

We currently do not know whether the post-course increment in self-efficacy is sustainable over time. A follow up study would be necessary to investigate this issue. Another direction for future studies is a cross-cultural comparison of educational interventions and their effects on ML self-efficacy, in order to better understand the impact of culturally diverse pedagogical approaches and identify ways to improve self-efficacy across the field.

### Limitations

This study is preliminary. The sample is small, and statistical results might be biased. Comparison conditions necessary for determining the specific effects of each course attribute were not included. Thus, the observed results may have been specific to factors such as the lecturer’s personality, the articulation of the ML course with other courses in the PT program, and the profile of students attending the institution. We believe that the opportunity to experience ML theory in a personally meaningful way had an especially positive effect on self-efficacy, although the differential effects of the experiential approach compared to an exclusively lecture-based version of the course must be tested. Whether the present results can be replicated with other educators in other contexts and will have an impact on future PTs’ practice remains to be seen. Additionally, the PTP-ML [3] and the framework [14] are both relatively new tools and their relevance to clinical practice has not been researched extensively. Future controlled studies could determine the essential components needed in educational interventions to promote the acquisition of ML knowledge and skills and, most importantly, positively affect clinical practice.

## Conclusions

The results suggested that the structured, early and active ML course is associated with important improvements in student’s knowledge and self-efficacy; it is thus now part of our fixed PT curriculum. Participants showed significant and important increases in their self-efficacy that appeared to be adequately grounded in their actual knowledge. Notably, after the course their level of self-efficacy was superior to that of experienced professionals who had had access only to conventional and unstructured contact with ML content. Because self-efficacy mediates the clinical application of knowledge and skills, systematic, active training in ML may help reduce the science-practice gap.

## Supplementary Information

**Additional file 1: Supplementary material.** Course structure, clinical case in final exam, and code book.

## Data Availability

The data analyzed during the current study are available from the corresponding author on request.

## Abbreviations

ML: Motor learning
PT: Physical therapy
PTs: Physical therapists
PTP-ML: Physical Therapists’ Perceptions of Motor Learning
SDC: Smallest detectable change
LOA: Limits of agreement
