# Supplementary material for: Testing a new active learning approach to advance motor learning knowledge and self-efficacy in physical therapy undergraduate education
Source: BMC Med Educ. 2021 Jan 19;21:62. doi: 10.1186/s12909-021-02486-1 (PMC7816454; doi:10.1186/s12909-021-02486-1)
Supplement: Supplementary file 1 — Additional file 1: Supplementary material. Course structure, clinical case in final exam, and code book. [file 12909_2021_2486_MOESM1_ESM.docx]

**Supplementary material: Course structure, clinical case in final exam, and code book**

**Structure of the Motor Learning Course**

| **Course objectives** | |
| --- | --- |
| Learning outcomes | By the completion of the course students will be able to:  (1) read, analyze and synthetize scientific papers related to motor learning science applied to clinical practice; (2) plan and execute motor skill acquisition programs applicable to rehabilitation patients. |
| **Course content** | |
| Basic concepts   - Definition of motor learning - Performance versus motor learning - Assessment of learning: retention and transfer - Habit versus skill - Cognitive versus motor skills - Classification of motor skills - Talent and effort, deliberate practice - Specificity of practice and generalizability - Forms of learning: explicit and implicit - Reinvestment - Stages of learning - Goal setting (SMART method) - Motivation and engagement - Performance measures to track progress | |
| Practice variables   - Instructions:   - Avoiding attentional overload and anxiety   - Instructions about the task and about movement performance   - External or internal focus of attention   - Demonstrations   - Manual help   - Equipment support - Types and Organization of practice:   - Task-specific   - Simulation   - Whole and part (simplification, segmentation and fractionization)   - Blocked, serial and random   - Constant and varied   - Massed and distributed - Feedback:   - Sensory and augmented   - Functions: information, motivation, reinforcement and guidance   - Timing, precision, and frequency effects   - Knowledge of performance vs knowledge of results   - Qualitative vs quantitative   - Descriptive vs prescriptive   - Fading schedules: summary, bandwidth, average, learner-requested | |
| Learning strategies   - Errorless learning (e.g. clinical protocol: constraint-induced movement therapy & shaping) - Analogy learning - Observational learning - Trial and error learning (e.g. clinical protocol: cognitive orientation to occupational performance) - Dual-task learning - Discovery learning - Movement imagery | |
| **Pedagogical methods** | |
| Lectures | Weekly lectures addressing the course contents |
| Team-based learning (TBL) | Three TBL sessions, one for each major instructional unit. Each session has the following structure:   1. Pre-class individual study (assigned readings) 2. Readiness assurance tests: Individual test, same test completed as a team, appeals, instructor clarifications 3. Application of concepts to clinical cases (by teams) |
| *Learning by Doing* project ([McLaughlin & Rogers, 2010](https://journals.sagepub.com/doi/pdf/10.1177/154193121005400802?casa_token=QUEwWBVHXaEAAAAA:iGyV5BLDAXeVulc6tzDhlctG-R7-QAXq7hxd1UeR4E_TXWltIBgguOhUv00_iH71yz7vV3bWxeZjjQ)) | Students learn about acquisition of motor skills by personally experimenting with it.  Activity components:  (1) Select a task/skill  Students choose an unfamiliar motor skill. Suggestions are provided:   - Dance (different styles of ballet, ballroom dances, street dance, etc.) - Sports (volleyball, basketball, football, soccer, cheerleading, etc.) - Martial arts (Karate, Judo, Tae-kwon-do, Aikido, etc.) - Yoga - Drawing - Calligraphy - Musical instruments (guitar, percussion, etc.) - Circus skills - Sculpture - Crochet - Origami - Skating     (2) Set a personal Specific, Measurable, Achievable, Realistic and Timely (SMART) performance goal for the skill acquisition experience ([Bovend’Eerdt, Botell, & Wade, 2009](https://journals.sagepub.com/doi/pdf/10.1177/0269215508101741?casa_token=VZjgyVEahB0AAAAA:JDclaAn1kyujEIu0HR5HUaYgV8hTvjBaGF8QEFNPshfYlVHJvwXlAUyUgYGTHUqgwIlK3md7_1NzXw)). The time frame for the SMART is 10 weeks of practice.  (3) Define performance (outcome) measures adequate to register the baseline level of performance and track progress over time, weekly.  (4) Plan practice sessions in regard to learning elements (instructions, practice structure and feedback), learning strategies (if a particular method is chosen) and forms of learning (implicit to explicit) ([Kleynen et al, 2018](https://www.tandfonline.com/doi/10.1080/09593985.2018.1483987))  (5) Practice the selected skill for 3 to 8 hours a week (to simulate the schedule of a patient in rehabilitation), for 10 weeks.  (6) Write biweekly skill acquisition log (documentation of all aspects in the process, see details under course requirements) |
| **Course requirements** | |
| Skill acquisition and reflection log | Written report that accompany the learning by doing project. Requires reporting through 5 web forms (every other week). about qualitative and quantitative changes, errors, difficulties, apparent plateaus, set-backs, frustrations, and so on. Everyone’s reports will be periodically in the classroom. |
| Skill acquisition demonstration | After 10 weeks, students demonstrate their skill to the class, either live or on video. They are required to show the complete process, from the determination of their SMART goal to the choices of elements (practice structure, instructions, feedbacks), weekly progress (results of your performance measures) and the final result. They use their theoretical knowledge combined with personal experience to analyze what worked and what didn’t and advise future learners. |
| Final *Learning by Doing* paper | A 5-page essay with a review of relevant scientific studies pertaining to the acquisition of the chosen skill, an overall report of the learning experience, and an analysis of how the theory fits (or doesn’t) the experience. |
| Partial exams | Seven multiple choice tests with 10 questions each, taken every other week |
| Final exam | 42 multiple-choice questions, including 10 questions based on a clinical case. |
| **Course references** | |
| Textbooks   - Schmidt RA, Lee TD, Winstein C, Wulf G, Zelaznik HN. Motor control and learning: A behavioral emphasis. United States of America: Human Kinetics; 2018. - Schmidt RA, Wrisberg CA. Motor learning and performance: A situation-based learning approach. United States of America: Human Kinetics; 2008.   Mandatory reading   - Bovend'Eerdt TJ, Botell RE, Wade DT. Writing SMART rehabilitation goals and achieving goal attainment scaling: a practical guide. Clinical rehabilitation. 2009;23(4):352-361. - Kleynen M, Beurskens A, Olijve H, Kamphuis J, Braun S. Application of motor learning in neurorehabilitation: a framework for health-care professionals. Physiother Theory Pract. 2018; doi:[10.1080/09593985.2018.1483987](https://doi.org/10.1080/09593985.2018.1483987) - Pollock CL, Boyd LA, Hunt MA, Garland S J. Use of the challenge point framework to guide motor learning of stepping reactions for improved balance control in people with stroke: a case series. Physical therapy. 2014;94(4):562-570. - Lanier VM, Lang CE, Van Dillen LR. Motor skill training in musculoskeletal pain: a case report in chronic low back pain. Disability and rehabilitation. 2019;41(17):2071-2079. - Missiuna C, Mandich AD, Polatajko H J, Malloy-Miller T. Cognitive orientation to daily occupational performance (CO-OP) part I-theoretical foundations. Physical & occupational therapy in pediatrics. 2001;20(2-3):69-81. - Gordon AM, Charles J, Wolf SL. Methods of constraint-induced movement therapy for children with hemiplegic cerebral palsy: development of a child-friendly intervention for improving upper-extremity function. Archives of physical medicine and rehabilitation. 2005;86(4):837-844.   Optional texts   - McLaughlin AC, Rogers WA. Learning by doing: Understanding skill acquisition through skill acquisition. Proc Hum Factors Ergon Soc. 2010;1:657–61. - Bjork EL, Bjork RA. Making things hard on yourself, but in a good way: Creating desirable difficulties to enhance learning. Psychology and the real world: Essays illustrating fundamental contributions to society. 2011;2:59-68. - Kleim JA, Jones TA. Principles of experience-dependent neural plasticity: implications for rehabilitation after brain damage. J Speech Lang Hear Res. 2008;51(1):S225-S239. | |

***Patient case in the final exam***

A PT is responsible for the rehabilitation of a soccer player who has experienced a hamstring strain during a game. According to the PT’s assessment, the strain happened because of inadequate trunk movement during a penalty kick (registered in video). The patient set the goal of doing penalty kicks without risky movement patterns to avoid recurrence of injury.

The patient is a 35-year-old male who began playing soccer at 25. His general physical fitness is excellent. He is the best player of the team at the company where he works as an accountant. The team has been taking part in many tournaments lately and does not have a coach. He tells the PT that he gets very anxious about the high performance expectations everyone else puts on him. He describes himself as introspective, methodical, and highly self-critical.

The PT worries that the patient will suffer from “paralysis by analysis” when he feels pressured at a game. Paralysis by analysis can occur when performance pressure increases the anxiety about performing correctly; this, in turn, enhances the attention paid to the step-by-step control of an action that otherwise would be performed automatically, spontaneously, and flexibly. Because of this cognitive “reinvestment,” old (and in this case, potentially injury-causing) movement patterns may emerge.

Therefore, the PT wants to favor *implicit learning* of safe kicking techniques (see the framework by Kleynen et al., 2018, below). Implicit learning is associated with a lower risk of cognitive reinvestment and paralysis by analysis.


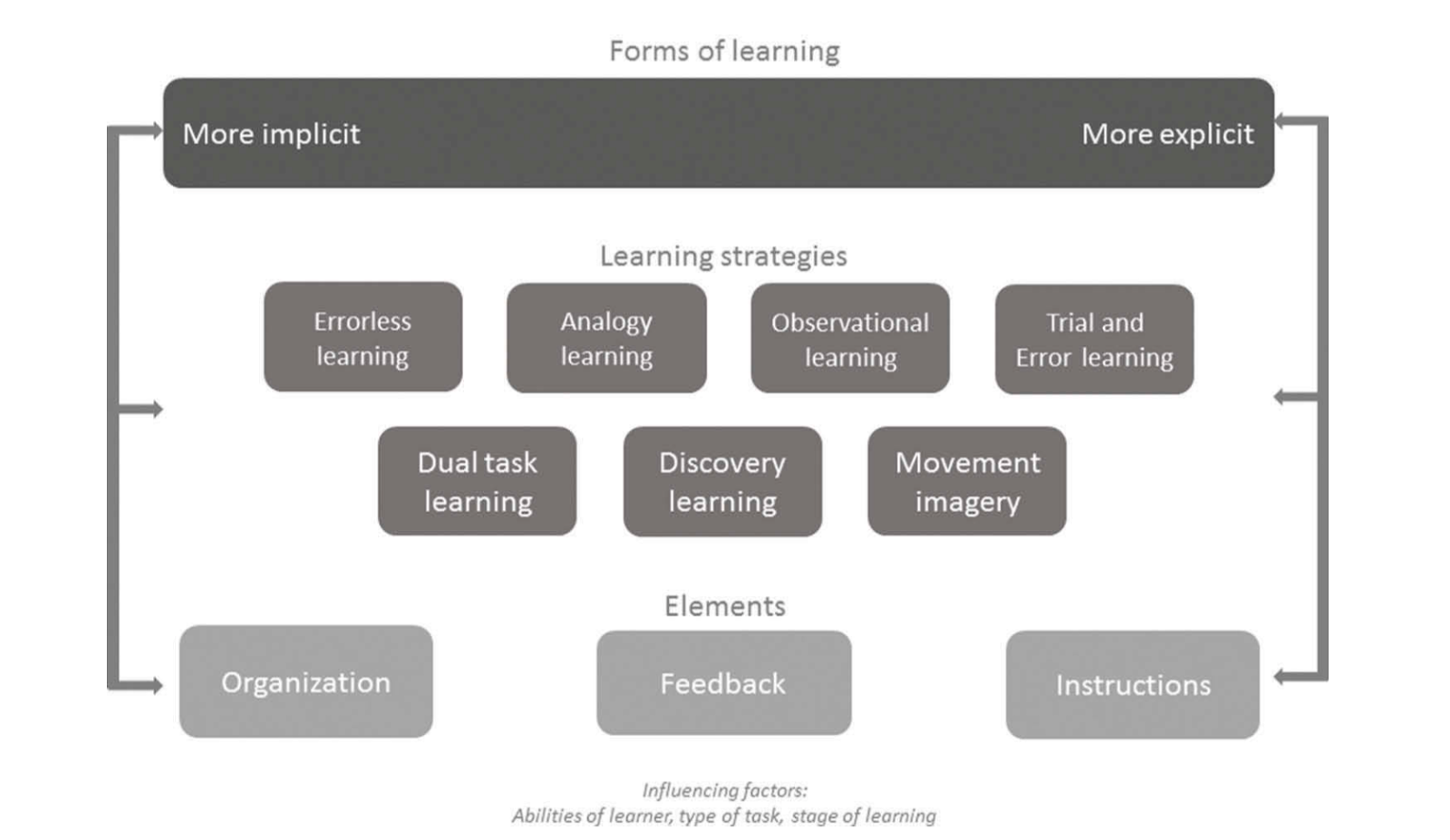


Reference: Kleynen M, Beurskens A, Olijve H, Kamphuis J, Braun S. Application of motor learning in neurorehabilitation: A framework for health-care professionals. *Physiotherapy Theory and Practice*, 2018;00:1-19.

Below, classify the treatment idea as Adequate (A) or Inadequate (I) for this patient’s case.

| **Treatment Idea** | **Answer**  **(A or I)** |
| --- | --- |
| 1. During the initial phase of practice, the PT will give frequent feedback about the level of activation of the hamstrings, quadriceps, abdominis, and latissimus dorsi to facilitate progressive increase of skill and minimize errors. Feedback frequency will be progressively reduced. |  |
| 2. During the initial phase of practice, while pain still exists in the hamstring, the PT will teach the patient how to practice movement imagery. |  |
| 3. During the initial phase of practice, the PT will deconstruct the kick into its basic components. The patient will be asked to practice each component separately. Later, components will be chained together progressively, until the kick can finally be practiced as a whole. |  |
| 4. During the initial phase of practice, the PT will use balls that are larger and lighter than an official soccer ball, making it easier for the patient to kick with the right technique. As the patient acquires the ability to kick correctly, smaller and heavier balls (more similar to an official soccer ball) will be used. |  |
| 5. During the intermediate and advanced phases of practice, the PT will use distractions while the patient performs kicks. For example, the patient may be asked to tell a story, remember items off a list, or perform calculations. |  |
| 6. Throughout practice, the PT describes the goal movement patterns with metaphors and analogies, such as the following: “Imagine that your leg moves as a whip,” or “Imagine that you are as stable as a samurai.” |  |
| 7. Throughout practice, the PT prioritizes feedback with knowledge about the action results instead of knowledge about how the patient performed the action. When information about the movement technique is needed, the PT seeks for analogies that inform the patient of the global movement pattern. |  |
| 8. Throughout practice, the PT stimulates the patient’s use of self-generated information to create independence. The PT frequently asks how he felt during the movement, and what he may do differently to perform better at the next trial. |  |
| 9. Throughout practice, the PT avoids verbal instructions about how to perform the proper kick. Instead, videos of the patient performing the adequate kick pattern (when performed not under pressure) are used as a demonstration. |  |
| 10. Throughout practice, the PT stimulates the patient to focus on the movement of the ball toward the goal box instead of focusing on his own body while he kicks. |  |
| 11. During the later phase of practice, the PT structures the sessions so that each kick style is practiced in a block. This way, the patient can focus on and recognize the mistakes in his movement pattern. |  |

**Code Book - Definitions of learning concepts**

1. **Stages of skill acquisition**

Three relatively distinct and sequential stages of the learning process

Refers to three relatively distinct and sequential stages of the learning process: 1) verbal-cognitive (primary concern is to understand what needs to be done, mostly through interpretative, declarative processes)¹, 2) associative (learners focus on refining the skill with subtle performance adjustments, movements become more consistent and improvements more gradual)¹, 3) autonomous (the motor skill can be performed with little deployment of attentional resources and less interference from other simultaneous activities)².

**2. Goal setting, meaningful goals**

Personal skill-related goals that are salient and challenging for the individual

Refers to what the individual wants to be able to do after a period of practice. Goals may refer to the results of the movement or activity (e.g. winning the game) or to the quality of movement patterns (e.g. correct ballet move)³.

**3. Active involvement/problem solving**

Engagement of the learner in finding motor solutions to achieve the task goal

Refers to creative ways to overcome difficulties during practice.

**4. Challenge/task difficulty**

The interaction between task demands and the skill level of the individual

Refers to the interaction between task demands and the skill level of the individual. Excessive task demands can reduce performance.

**5. Learning mechanism**

Explicit and/or implicit processes of skill acquisition

Explicit (or conscious) mechanism refers to motor learning that takes place through acquisition of verbal knowledge (i.e. facts and rules) about the movement. The individual is aware of what he or she is learning.

Implicit (or non-declarative, procedural) mechanism refers to motor learning with no or minimal increase in verbal knowledge (e.g., facts and rules) about of movement performance and without the individual’s awareness⁴.

**6. Classification of motor skills/type of task**

One or two-dimensional classifications of skills

Refers skills being classified according to a given dimension (e.g. discrete/serial/continuous, open/closed, simple/complex, fine/gross, self-paced/externally-paced) or according to the combination of the two dimensions of action requirements (body transport /object manipulation) and environmental demands (regulatory variability/context variability)¹.

**7. Content and type of feedback**

Information contained in feedback and feedback sources

Refers to the classifications of feedback content as knowledge of performance (informs about the quality of the movement) or knowledge of results (informs about the success the actions in relation to the intended goal)⁵, as qualitative or quantitative, as descriptive or prescriptive. Feedback type can be intrinsic (internal sources) or extrinsic (augmented, from external sources)⁶.

**8. Frequency and/or timing of feedback**

The schedule of feedback provision

Refers to the scheme of feedback provision relative to the practice trials. Frequency may vary between giving feedback after every trial to only after a number or trials (summary or average feedback) or only after trials that exceed a certain threshold of error (bandwidth feedback). Timing can vary between giving feedback during movement (concurrently) or immediately after (terminally) or some time after the performance of the movement (delayed)⁵.

**9. Focus of attention (internal/external)**

Direction of attentional resources

Refers to the learner directing his/her attention internally (inward to the body and the underlying processes of the movement itself) or externally (outwards on the goal or the effect of the movement)⁵.

**10. Task breakdown (whole/part)**

Practice of tasks as a whole or by parts

Refers to either practicing a complex task as a whole or practicing it by parts. The types of partial practice include fractionalization (one or more parts of a skill are practiced separately)³, segmentation (progressive partial practice: a part of a target skill is practiced for some time, then the second part is added to the first part, and the two are practiced together, until the entire target skill is practiced) and simplification (the complexity of some aspect of the target task is reduced, e.g. by practicing in slow motion or using a larger ball to serve in tennis)³.

**11. Amount of practice**

Volume of practice

Refers to the number of repetitions or the total duration of practice.

**12. Practice variability (constant/variable)**

Change or constancy in the way the skill is practiced

Refers to repeating only one variation of a particular skill (constant practice) or practicing several variations of the skill (varied practice)⁷.

**13. Order of practice (random/serial/block)**

The sequencing of practice trials

Refers to practicing the same skill (or skill variation) repeatedly (blocked practice), or different skills (or skill variations) in a predefined sequence (serial practice), or practicing a variety of skills (or skill variations) in random order (random practice)⁷.

**14. Practice distribution (massive or distributed)**

The spacing of practice sessions

Refers to practicing a skill with no or very little spacing between sessions for rest (massed practice) or to alternating periods of practice with periods of rest (distributed practice).²

**15. Specificity of practice**

Practice conditions should match the expected performance conditions

Refers to the principle that the transfer between the conditions of practice and later performance in real-world settings depends upon the similarity between the two⁷.

**16. Positive reinforcement**

Use of rewards to increase in the likelihood that the response will be repeated

Refers to a reward (such as praise for good performance) that is obtained after an action, increasing the possibility that the learner will repeat that same action under similar circumstances⁶.

**17. Task-oriented/task-specific training**

Goal-directed, functional task practice for acquisition or re-acquisition of skills

Refers practicing real-life tasks (such as reaching and grasping objects) to promote skill acquisition instead of practicing isolated movement components (such as flexion/extension, abduction/adduction and rotation of upper extremity joints) to reduce impairments.

**18. Mental practice**

Learning strategy (from conceptual framework)^5^

Refers to practicing trough imagining oneself performing the skilled movement (movement imagery in the first- or third-person perspective) without actually physically performing the movement^5^.

**19. Manual guidance**

Non-verbal, physical instruction through movement

Refers to the instructor moving the learner's body through the fundamental movement pattern³.

**20. Observational learning/modeling**

Learning strategy (from conceptual framework)^5^

Refers to learning by observing a movement: the observer determines the key spatial and/or temporal features of the task through observation, and/or is directed to these features by the demonstrator/ therapist^5^.

**21. Dual-task learning**

Learning strategy (from conceptual framework)^5^

Refers to learning of a skill while simultaneously performing another task, which can be a motor or cognitive task but must be attention demanding^5^.

**22. Discovery learning**

Learning strategy (from conceptual framework)^5^

Refers to learning without guidance or feedback from another person or information source^5^.

**23. Trial and error learning**

Learning strategy (from conceptual framework)^5^

Refers to learning by repeatedly attempting to perform a task during which the learner detects errors and corrects them^5^.

**24. Errorless learning**

Learning strategy (from conceptual framework)^5^

Refers to learning facilitated by constraining the learning experience (e.g. providing instructions and corrective feedback, decreasing the difficulty of task, changing the environment) so that very few errors occur^5^.

**25. Analogy learning**

Learning strategy (from conceptual framework)^5^

Refers to learning facilitated by metaphors: the complex structure of the to-be-learned skill is integrated into a simple metaphor that the learner is provided with^5^.

**26. Transfer of learning**

The influence of learning one skill on the performance of another skill

Refers to the gain or loss of a person's proficiency in one task as a result of previous practice or experience in another similar task¹.

**Conceptual mistakes**

Inaccurate or confused application of concepts

This code can be applied in addition to all the numbered codes in case the student uses a given concept in ways that are inconsistent with its definition.

**REFERENCES**

1 Schmidt RA, Wrisberg CA. Preparing for the Learning Experience. In: Schmidt RA, Wrisberg CA. Motor learning and performance: A situation-based learning approach. United States of America: Human Kinetics;2008. p. 189-216.

2 Zwicker JG, Harris SR. A reflection on motor learning theory in pediatric occupational therapy practice. Canadian Journal of Occupation Therapy. 2009;76(1):29-37.

3 Schmidt RA, Wrisberg CA. Supplementing the Learning Experience. In: Schmidt RA, Wrisberg CA. Motor learning and performance: A situation-based learning approach. United States of America: Human Kinetics;2008. p. 219-252.

4 Kleynen M, Braun SM, Bleijlevens MH, Lexis MA, Rasquin SM, Halfens J, et al. Using a Delphi Technique to Seek Consensus Regarding Definitions, Descriptions and Classification of Terms Related to Implicit and Explicit Forms of Motor Learning. PLoS ONE [Internet]. 2014;9(6):e100227. Available from: doi:10.1371/journal.pone.0100227

5 Kleynen M, Beurskens A, Olijve H, Kamphuis J, Braun S. Application of motor learning in neurorehabilitation: a framework for health-care professionals. Physiother Theory Pract [Internet].2018;00(00):1–19. Available from: https://doi.org/10.1080/09593985.2018.1483987

6 Schmidt RA, Wrisberg CA. Providing Feedback During the Learning Experience. In: Schmidt RA, Wrisberg CA. Motor learning and performance: A situation-based learning approach. United States of America: Human Kinetics;2008. p. 284-318.

7 Schmidt RA, Wrisberg CA. Structuring the Learning Experience. In: Schmidt RA, Wrisberg CA. Motor learning and performance: A situation-based learning approach. United States of America: Human Kinetics;2008. p. 256-280.
